# Genetic Analysis of *Leishmania donovani* Tropism Using a Naturally Attenuated Cutaneous Strain

**DOI:** 10.1371/journal.ppat.1004244

**Published:** 2014-07-03

**Authors:** Wen Wei Zhang, Gowthaman Ramasamy, Laura-Isobel McCall, Andrew Haydock, Shalindra Ranasinghe, Priyanka Abeygunasekara, Ganga Sirimanna, Renu Wickremasinghe, Peter Myler, Greg Matlashewski

**Affiliations:** 1 Department of Microbiology and Immunology, McGill University, Montreal, Canada; 2 Seattle Biomedical Research Institute, Seattle, Washington, United States of America; 3 Department of Parasitology, University of Sri Jayewardenepura, Gangodawila, Sri Lanka; 4 Teaching Hospital Anuradhapura, Anuradhapura, Sri Lanka; 5 Dermatology Unit, National Hospital of Sri Lanka, Colombo, Sri Lanka; 6 Microbiome and Disease Tolerance Centre, McGill University, Montreal, Canada; University of British Columbia, Canada

## Abstract

A central question in *Leishmania* research is why most species cause cutaneous infections but others cause fatal visceral disease. Interestingly, *L. donovani* causes both visceral and cutaneous leishmaniasis in Sri Lanka. *L. donovani* clinical isolates were therefore obtained from cutaneous leishmaniasis (CL-SL) and visceral leishmaniasis (VL-SL) patients from Sri Lanka. The CL-SL isolate was severely attenuated compared to the VL-SL isolate for survival in visceral organs in BALB/c mice. Genomic and transcriptomic analysis argue that gene deletions or pseudogenes specific to CL-SL are not responsible for the difference in disease tropism and that single nucleotide polymorphisms (SNPs) and/or gene copy number variations play a major role in altered pathology. This is illustrated through the observations within showing that a decreased copy number of the A2 gene family and a mutation in the ras-like RagC GTPase enzyme in the mTOR pathway contribute to the attenuation of the CL-SL strain in visceral infection. Overall, this research provides a unique perspective on genetic differences associated with diverse pathologies caused by *Leishmania* infection.

## Author Summary

Visceral leishmaniasis is one of the most lethal parasitic diseases, and the mechanisms that govern its survival in visceral organs are not understood. Here, we obtained an atypical cutaneous *Leishmania donovani* clinical isolate from Sri Lanka and compared it to a typical visceral disease causing clinical isolate. Through whole genome sequencing, bioinformatics analysis, experimental infection in mice and functional genomic analysis, this study provides novel information on what differentiates a deadly visceral strain from a benign cutaneous strain. Results indicate that the ability of *Leishmania* parasites to cause visceral or cutaneous leishmaniasis may be determined by mutations or amplification of a few genes, or combinations of these factors. Overall, this work contributes to the understanding of parasite virulence and may help guide disease control efforts.

## Introduction

Leishmaniasis is a neglected tropical disease present in 98 countries, with over 350 million people at risk of infection and is caused by *Leishmania* protozoan parasites transmitted by infected sand flies [1], [2]. Visceral leishmaniasis is the most serious form of this disease and it is among the most lethal parasitic infections after malaria. Cutaneous leishmaniasis in comparison causes skin lesions which usually self-heal. Over 20 *Leishmania* species can infect humans; however only the *Leishmania donovani* complex including *L. infantum* cause the vast majority of visceral leishmaniasis cases worldwide [2], [3].

In Sri Lanka, an atypical *L. donovani* (strain MON-37) has been responsible for thousands of cutaneous leishmaniasis cases in the past decade [4], [5], [6]. This is of considerable interest because *L. donovani* typically causes visceral leishmaniasis in Asia and Africa. Visceral leishmaniasis is rare in Sri Lanka, with the first recorded case only in 2007 [7] and only 4 cases of autochthonous visceral leishmaniasis reported so far. We have recently demonstrated that the visceral leishmaniasis-causing strain is also *L. donovani* MON-37 [8]. It is however unknown whether the same or different sub-strains of *L. donovani* MON-37 are responsible for visceral and cutaneous leishmaniasis in Sri Lanka.

Among the most important questions in *Leishmania* research is why some species remain at the site of the sandfly bite and cause cutaneous infections and others metastasize to the internal organs where they cause visceral disease. By comparing genomes of *Leishmania* species which cause different pathology, we previously identified several *L. donovani* genes including A2 and Ldbpk_280340 which are required for visceral organ tropism [9], [10]. However, comparing genomes of different *Leishmania* species is insufficient to fully define determinants of disease tropism and pathology because of their evolutionary distance, introducing genetic changes unrelated to human pathology [3]. A more effective strategy is to compare genomes of closely related *Leishmania* isolates of the same species that cause different human pathologies. We therefore undertook a phenotypic and genotypic analysis of *L. donovani* clinical isolates derived from cutaneous and visceral leishmaniasis patients in Sri Lanka. Characterization of these *L. donovani* clinical isolates provides invaluable insight into the etiology of visceral and cutaneous leishmaniasis in Sri Lanka and provides unique insight into the genetic basis of visceral leishmaniasis.

## Results

### Infection of BALB/c mice with the CL-SL and VL-SL *L. donovani* strains

The cutaneous leishmaniasis *L. donovani* isolate (CL-SL) from Sri Lanka was derived from a skin lesion as detailed in methods and the visceral leishmaniasis *L. donovani* strain (VL-SL) has recently been reported [8]. We initially re-sequenced the 6-phosphogluconate dehydrogenase (6PGDH) isoenzyme gene from the CL-SL and VL-SL isolates to confirm they were both *L. donovani* MON-37 (Fig. S1).

**Figure 1:**
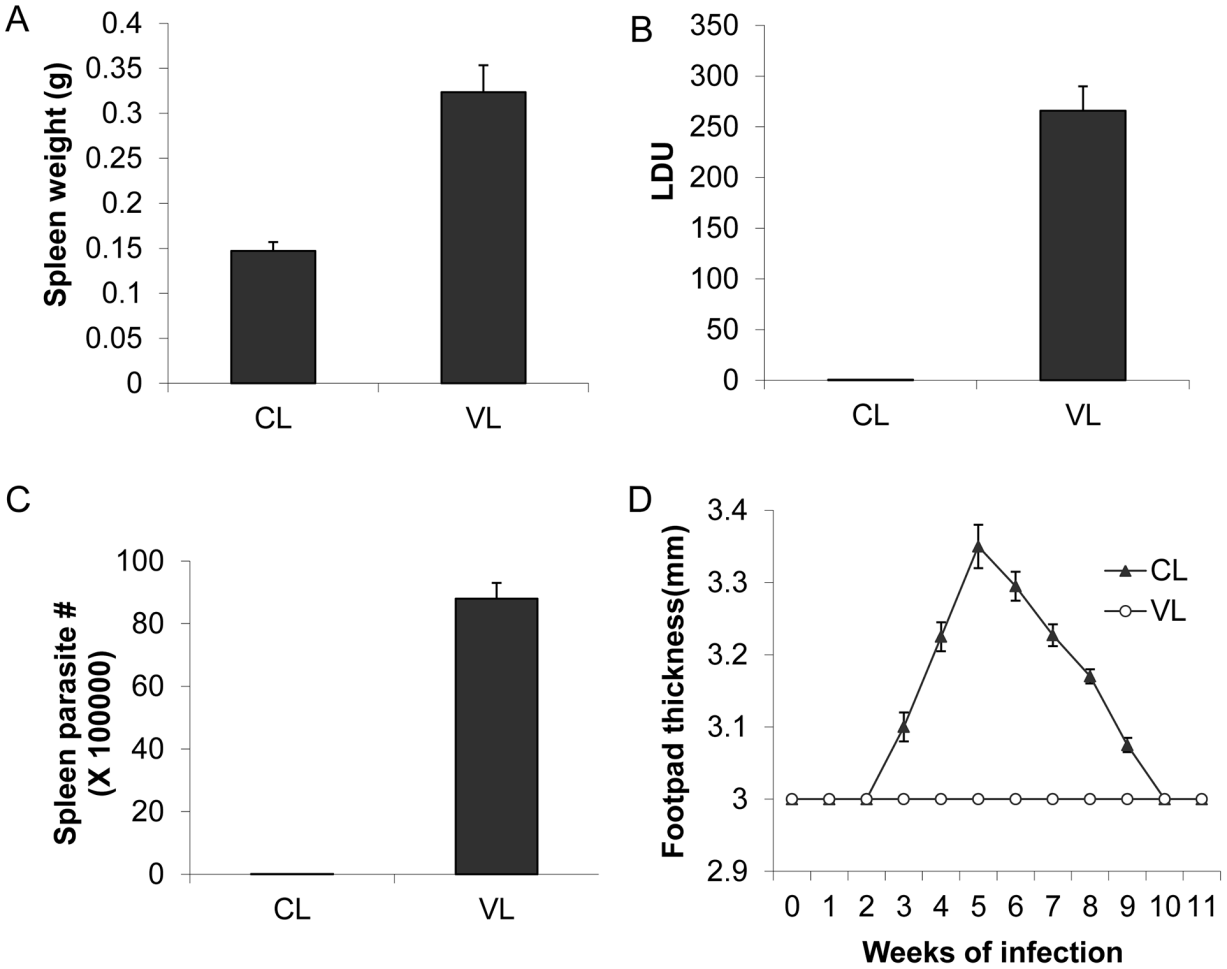
The *L. donovani* CL-SL (CL) and VL-SL (VL) strains cause different pathology in BALB/c mice. For visceral infection, BALB/c mice (5 mice/group) were infected in the tail vein with 5×10^7^ stationary phase promastigotes and 4 weeks after infection, mice were examined for spleen weight and parasite burden in the liver (Leishman Donovan Units: LDU) and spleen (parasite numbers) as determined by limiting dilution of spleen homogenates. The VL-SL strain (VL) infection in mice resulted in splenomegaly (**Panel A**) and high levels of infection in the liver (**Panel B**) and spleen (**Panel C**) compared to the CL-SL strain (CL) that caused negligible infection levels in the liver and spleen. Values plus standard error are shown. **Panel D**, cutaneous infections, BALB/c mice (5 mice/group) were injected subcutaneously in the rear footpad with 5×10^6^ stationary phase promastigotes and footpad lesion development was measured over 11 weeks.

These isolates were then compared with respect to their ability to cause disease when experimentally introduced into mice. BALB/c mice were injected in the tail vein with CL-SL or VL-SL to compare their ability to cause visceral infections and subcutaneously with CL-SL or VL-SL in the rear footpad to assess cutaneous infection. Liver and spleen parasite burdens were determined 4 weeks after visceral infection, and footpad swelling was monitored for 11 weeks following cutaneous infections. As shown in Fig. 1 A–C, mice injected with the VL-SL isolate had high levels of visceral infection in the liver and spleen. Mice injected with the CL-SL isolate had very little detectable visceral organ infection, demonstrating that the CL-SL parasite has lost the ability to survive in visceral organs (Fig. 1B, C). With respect to cutaneous infections, both the CL-SL and VL-SL isolates demonstrated low virulence; however, the CL-SL isolate was able to induce transient footpad swelling while the VL-SL isolate was unable to do so (Fig. 1D). Although the CL-SL isolate induced more footpad swelling than the VL-SL isolate, this was not associated with a significant increase in parasite number (Fig. S2) and therefore was likely due to a stronger inflammatory response.

Overall, these results conform to Koch's postulates demonstrating that cutaneous and visceral leishmaniasis are caused by distinct but closely related strains of *L. donovani*. These include 1) isolates were obtained directly from cutaneous or visceral sites of human infection; 2) isolates could be cultured and identified by isoenzyme gene sequencing; 3) isolates induced similar pathologies in experimental animals as in humans; and 4) the same strains could be re-isolated from the experimental infection site. Since the mouse infection phenotypes were similar to the human infections, this would argue that these isolates are representative of the cutaneous and visceral disease causing strains circulating in Sri Lanka.

### Genome sequencing

The above experimental infections demonstrated that the CL-SL and VL-SL *L. donovani* isolates were phenotypically distinct and therefore provided strong justification for comparing their genomes. Whole genome sequencing was performed using the Illumina GAIIx next generation sequencer >200× coverage as detailed in supplementary methods (Text S1). The sequences of the CL-SL and VL-SL genomes were aligned and compared against the *L. donovani* reference strain BPK282A1 originating from Nepal [11], [12].

There were no gene deletions detected in either genome. However, coverage analysis of gDNA-seq reads in 10 kb segments from the CL-SL and VL-SL isolates indicated that there were nine regions with copy number variations some of which contained several genes (Table 1). Those higher in the VL-SL isolate included Ldbpk_111220.1 (ABC transporter) repeat cluster, LdbpK_161030.1 thru LdbpK_161110.1 hypothetical gene cluster, and LdbpK_200120.1 (phosphoglycerate kinase B, cytosolic and an rRNA locus in chromosome 27. The species-specific A2 multi-gene family coding regions and the flanking 5′ and 3′ A2rel genes [13] were assembled and examined manually because they are incorrectly assembled in the reference genome BPK282A1. Aligning genomic DNA reads against the reconstructed A2 region revealed the presence of more copies of the A2 genes in the VL-SL strain than the CL-SL strain (Table 1). The CL-SL isolate has higher copy number in a region of chromosome 23 which contains ABC thiol transporters (MRPA) H-region (LdbpK_230290.1), terbinafine resistance locus protein (yip1; LdbpK_230280.1) and regions of chromosome 1 (eukaryotic initiation factor 4a, putative), chromosome 19 (glycerol uptake proteins) and chromosome 29 (hypotheticals).

**Table 1:** Gene copy number variation between the VL and CL isolates.

Chr	Tile Start	Tile End	Tile VL/CL ratio	Gene VL/CL ratio	Gene count	GeneID	Product Names
1	240001	250001	0.88	0.54	1	LdBPK_010790.1	eukaryotic initiation factor 4a, putative
11	500001	510000	2.61	2.94	1	LdBPK_111220.1	ABC transporter-like protein
16	370001	420000	1.90	2.14	9	LdBPK_161030.1	(Hypothetical protein) Repeat cluster
				1.90		LdBPK_161040.1	hypothetical protein
				1.90		LdBPK_161050.1	hypothetical protein
				1.77		LdBPK_161060.1	hypothetical protein
				1.94		LdBPK_161070.1	hypothetical protein
				2.00		LdBPK_161080.1	hypothetical protein
				1.97		LdBPK_161090.1	hypothetical protein
				1.87		LdBPK_161100.1	hypothetical protein
				1.87		LdBPK_161110.1	hypothetical protein
19	560001	570000	0.55	0.49	2	LdBPK_191310.1	glycerol uptake protein, putative
				0.56		LdBPK_191320.1	glycerol uptake protein, putative
20	30001	40000	1.19	2.53	1	LdBPK_200120.1	phosphoglycerate kinase B, cytosolic
22	300738	322483	NA	1.83		LdBPK_220670.1	A2 and A2rel repeat cluster*
23	80001	100000	0.46	0.42	5	LdBPK_230250.1	ABC-thiol transporter (MRPA) H-region
				0.43		LdBPK_230260.1	hypothetical protein, conserved
				0.38		LdBPK_230270.1	hypothetical protein, conserved
				0.43		LdBPK_230280.1	terbinafine resistance locus protein (YIP1)
				0.46		LdBPK_230290.1	ABC-thiol transporter (MRPA)
27	1000001	1024085	1.34	NA	4+	Multiple rRNAs	rRNA locus
29	710001	720000	0.55	0.36	2	LdBPK_291620.1	hypothetical protein
				0.35		LdBPK_291630.1	hypothetical protein

* The A2-A2rel multi-gene family coding region was manually examined because this region is incorrectly assembled in the reference *L. donovani* BPK282A1 genome due to the repetitive sequences in A2 genes.

Chr: Chromosome.

### Single nucleotide polymorphism (SNP) and indel analysis

Differences identified between the CL-SL, VL-SL isolates and the reference *L. donovani* (BPK282A1) genome are summarized in Table 2. More than 80% of the differences are common to VL-SL and CL-SL revealing that the Sri Lanka isolates are more closely related to each other than to the reference *L. donovani* strain from Nepal. Furthermore, although the majority of variants are homozygous, about 20% of variants are heterozygous. As expected, SNPs accounts for the vast majority of variants which occur mostly in the intergenic regions, while about 20% of the SNPs are located in coding regions. Interestingly, there are over 70 pseudogenes resulting from frame shifts or stop codon variations which are in common to the Sri Lanka CL-SL and VL-SL isolates yet are functional in the reference *L. donovani* strain (Table 2). These genes therefore appear not to be essential or are not involved in virulence.

**Table 2:** Effects of SNPs and Indels on the genomes/proteomes of Sri Lanka *L. donovani* isolates.

Variants	Common to VL & CL		Unique to VL		Unique to CL	
	Homo^1^	Hete^2^	Homo^1^	Hete^2^	Homo^1^	Hete^2^
Frame shift	73	19	1	1	0	0
Stop Gained	15	4	0	1	0	1
Stop Lost	10	1	0	0	1	0
Start Lost	5	0	0	0	0	0
Codon Insertion	89	3	2	0	6	0
Codon deletion	103	1	7	3	7	1
Non Synonymous Coding	5615	370	70	72	70	47
Synonymous Coding	4077	208	40	38	60	32
Intergenic	28256	5,526	1857	1793	2107	2506
Total	38,243	6132	1977	1908	2251	2587

1 Homo (homozygous) indicates both alleles are different from reference strain.

2 Hete (heterozygous) indicates only one allele is different from reference strain.

Variations between the VL-SL or CL-SL isolates are among the most interesting, and those specific to the CL-SL isolate would be expected to cause loss of the ability to survive in visceral organs. Surprisingly, as summarized in Table 3, there are only 5 genes unequally affected in the CL-SL and VL-SL isolates by frame shift or altered stop site that could be considered to be pseudogenes. Two genes with frame shifts (LdBPK_312060.1 and LdBPK_320030.1) are only present as homozygous or heterozygous in the VL-SL isolate. There are no homozygous pseudogenes specific to the CL-SL isolate except LdBPK_311390.1 which only differs by 3 amino acids at the C-terminus when compared to its homolog from the VL-SL isolate.

**Table 3:** Genes unequally affected in the CL and VL isolates by frame shift or stop site change.

Chr	Position	Change	Gene ID	Normal Size(aa)	Affected Site(aa)	Effect	CL^2^	VL^2^	Gene product
30	579030	C/T	LdBPK_301640.1	696	334	Stop gained	Hete	wt	Hypothetical protein,
31	602800	C/A	LdBPK_311390.1	1486	1487	Stop lost^1^	Homo	wt	Hypothetical protein,
31	1000447	A/AG	LdBPK_312060.1	471	343	Frame shift	wt	Hete	Succinyl-diaminopimelate desuccinylase,
32	9103	C/CG	LdBPK_320030.1	367	102	Frame shift	wt	Homo	Hypothetical protein
32	174033	C/T	LdBPK_320480.1	939	701	Stop gained	wt	Hete	Hypothetical protein,

1. After the original stop lost, only three amino acids (LSH stop) are added.

2. Hete (heterozygous) indicates one allele different from reference strain (BPK282A1). Homo (homozygous) indicates both alleles different from reference strain. wt indicates both alleles the same as reference strain.

SNPs resulted in 117 (70 homo+47 hetero) non-synonymous and 92 (60 homo+32 hetero) synonymous changes specific to the CL-SL isolate (Tables 2, S1). Although the majority of these non-synonymous SNPs involve hypothetical proteins, 15 of the genes with homozygous mutations in the CL-SL isolate have putative functions (Table S1). Some of the non-synonymous SNPs in these 15 genes are located in functionally important regions of the genes described below. For instance, the non-synonymous change (G226D) in LdBPK_220120.1 phosphoinositide phosphatase is in a highly conserved region. Likewise, the non-synonymous change P147Q in LdBPK_171200.1 is in a highly conserved region of a putative 3-oxo-5-alphasteroid 4-dehydrogenase, responsible for the formation of dihydrotestosterone in higher eukaryotes [14]. LdBPK_252290.1 encodes a putative highly conserved DnaJ family protein that plays a role in protein translation, folding, translocation, and degradation [15]. LdBPK_322160.1 is a putative Rab GTPase family 2 (Rab2) protein involved in membrane trafficking [16]. LdBPK_341910.1 encodes a putative NAD dependent deacetylase which catalyzes NAD+-dependent protein/histone deacetylation and has been shown to regulate gene silencing, DNA repair, metabolic enzymes, and cellular life span [17]. LdBPK_366140.1 encodes a ras-like small GTPase-RagC which may be involved with mTOR regulated translation and cell growth [18]. SNPs in the non-coding region could also have an impact on protein levels since protein expression in *Leishmania* is regulated at the post-transcriptional level, including via sequences in the mRNA 3′UTR [19].

### Functional analysis of SNPs in conserved genes

**Figure 2:**
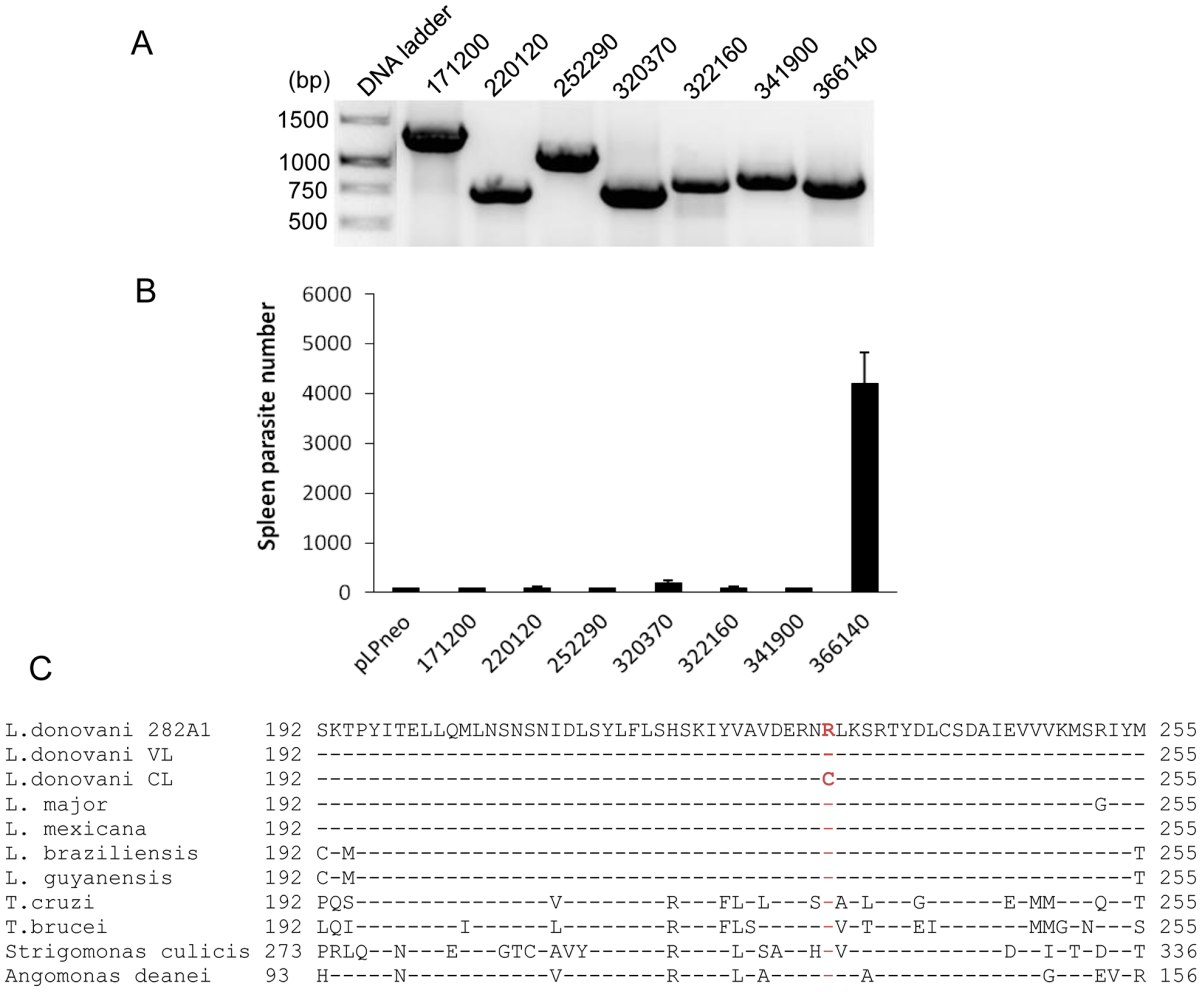
Expression of VL-SL *Rag C* gene (366149) in the CL-SL isolate increased its survival in the spleen. Genes from the VL-SL isolate were expressed in the CL-SL isolate, and spleen infection levels assessed in mice following intravenous infection. The VL-SL genes included are: LdBPK171200.1, steroid dehydrogenase; LdBPK220120, phosphoinositide phosphatase; LdBPK252290, DnaJ protein; LdBPK320370, hypothetical protein; LdBPK322160, rab-2a; LdBPK341900, NAD dependent deacetylase; LdBPK366149, *Rag C* (ras like GTPase C). **Panel A.** RT-PCR shows that all the corresponding VL-SL genes were transcribed in the transfected CL-SL *L. donovani* cells. **Panel B.** Infection levels in the spleen 4 weeks after infection. Values are mean plus standard error. **Panel C.** Alignments show that amino acid R231 in Rag C proteins is highly conserved in *Leishmania* and *Trypanosomes*.

We considered non-synonymous SNPs in the CL-SL isolate of potential interest if the sequence differed from both the VL-SL isolate and the reference *L. donovani* strain and if the SNP appeared to be in a functionally important part of the gene as described above. To determine the potential impact of CL-SL specific SNPs on visceral infection and to complement potential functional defects, we cloned 7 corresponding genes from the VL-SL isolate described above, transfected them into the CL-SL isolate and measured the survival of the transgenic parasites in the spleen 4 weeks after infection. All of the CL-SL specific SNPs were verified by Sanger sequencing prior to transfection and their expression in the transfected CL-SL isolate verified by RT-PCR (Fig. 2A). As shown in Fig. 2B, only transfection of the VL-SL *Rag C* gene into the CL-SL isolate significantly increased infection levels in the spleen. It is noteworthy that the increased infection levels in separate experiments ranged from a 5 to 40 fold and were statistically significant revealing that expression of the VL-SL *Rag C* gene in CL-SL increased survival in the visceral organs.

The *Rag C* gene (LdBPK_366140.1) in the CL-SL isolate has a homozygous non-conservative amino acid substitution (R231C). As shown in Fig. 2C, the R231C polymorphism is in a highly conserved amino acid across trypanosomatids. RagC is a small ras-like GTPase involved in the activation of the target of the mammalian rapamycin (mTOR) protein in higher eukaryotes. The mTOR protein is a conserved serine-threonine protein kinase involved in multiple cellular processes including cell stress and proliferation [18]. There are 3 *TOR* genes (*TOR 1–3*) identified in the *Leishmania* genome and *TOR 1–2* are essential whereas *TOR3* is not essential for survival in the promastigote stage but is required for survival in macrophages and infected mice [20].

### Chromosome somy and transcriptional analysis

**Figure 3:**
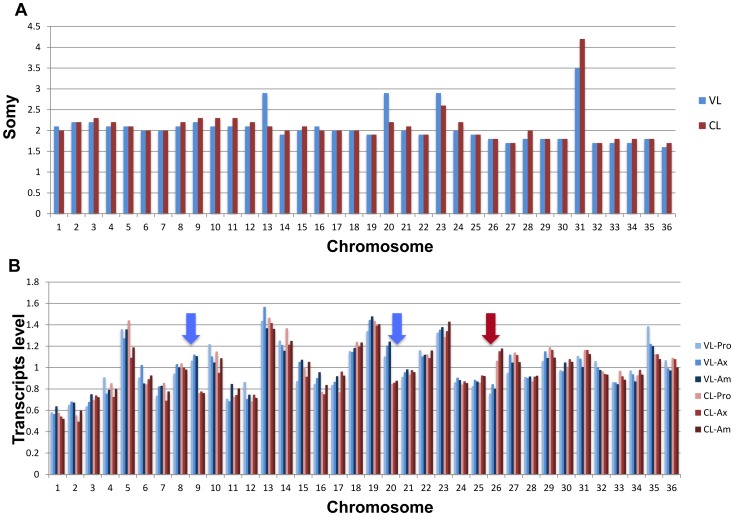
Chromosome copy number comparison between the VL-SL and CL-SL strains (Panel A). Read depth was scaled to give a value of 2 for disomic chromosomes. Comparison of chromosome transcript levels in the VL-SL and CL-SL strains (**Panel B**). Total RNA was prepared from promastigotes, axenic amastigotes and macrophages infected with the VL-SL and CL-SL strains. The cDNA libraries prepared from the RNAs were subjected to SL-sequencing and the cDNA coverage from each chromosome was normalized to the entire genome. Pro, axenic promastigotes; Ax, axenic amastigotes; Am, infected macrophage-derived amastigotes.

Chromosome somy can vary in different *Leishmania* species [11], [12], [21] and therefore it was necessary to determine if there were differences in chromosome somy that could account for the differences in pathology. As shown in Figure 3A, most of the chromosomes were disomic in both isolates except chromosome 31 which was tetrasomic as previously shown for *L. donovani* [11] and chromosome 23 which appears to be trisomic. With respect to differences between the isolates, chromosomes 13 and 20 appeared to be trisomic in VL-SL and diploid in CL-SL.

We next examined whether the differences in chromosome somy was reflected in differences at the transcript level. cDNA libraries were prepared using mRNA derived from axenic promastigotes (Pro), axenic amastigotes (Ax) and macrophage-derived amastigotes (Am) and entire chromosome transcript levels were compared between these two isolates (Fig. 3B). Several variations between chromosomes were apparent when the median mRNA levels (normalized to the entire genome) were compared; most appear to be unrelated to somy. For example, the mRNA levels are lower than average for many of the small chromosomes (except chromosome 5); chromosome 31 has average mRNA levels despite being tetrasomic; and the mRNA levels for chromosome 13 are high for both isolates even though chromosome 13 is trisomic in the VL-SL isolate. Despite the fact that chromosomes 9 and 26 are disomic in both isolates, the mRNA level for chromosome 9 is about 40% higher in the VL-SL isolate than in the CL-SL isolate; conversely, the mRNA level for chromosome 26 has at least a 30% increase in the CL-SL isolate. However, consistent with the increased somy, the median mRNA level for chromosome 20 has been increased nearly 1.5 times in the VL-SL isolate. Overall these data suggest that chromosome somy may not be a major factor in the different pathologies caused by these isolates since ploidy is not closely mirrored by transcript level.

When comparing the individual gene transcript levels in different life cycle stages, the vast majority of genes were expressed at similar levels. A list of the most differentially expressed genes with at least a 4 fold difference in mRNA levels in the amastigote infected macrophage stage is shown in Table 4. Genes with at least a 2 fold difference are shown in Table S2 for genes more highly expressed in the VL-SL isolate and Table S3 for genes more highly expressed in the CL-SL isolate. Two gene families identified in Table 4, the ABCA3 and A2 gene families, also have higher gene copy numbers as shown in Table 1. One of the rate limiting enzymes in the glycolysis pathway, glycosomal phosphoglycerate kinase (Ldbpk_200110.1) and two hypothetical proteins were also highly up-regulated in the VL-SL strain. The CL-SL isolate had higher mRNA levels of a ribosomal subunit protein and a putative adaptin protein. Overall, these mRNA level differences could impact energy production, protein synthesis and, with respect to A2, visceral organ survival.

**Table 4:** Genes with more than 4 fold change in transcript levels between the CL-SL and VL-SL isolates.

Transcripts	Gene ID	Folds change			Gene Product
		Am	Ax	Pro	
**Up in VL**	LdBPK_221230.1	21.0	19.1	17.2	hypothetical protein, conserved, mak-16-like RNA binding protein, putative
	LdBPK_361170.1	5.0	3.7	3.4	hypothetical protein, conserved
	LdBPK_200110.1	4.8	3.7	5.5	phosphoglycerate kinase C, glycosomal (PGKC)
	LdBPK_111230.1	4.5	5.5	2.7	ATP-binding cassette protein subfamily A, member 3, putative (ABCA3)
	LdBPK_220670.1	4.3	2.5	2.4	A2 protein
**Up in CL**	LdBPK_365490.1	4.7	4.4	4.5	adaptin, putative
	LdBPK_303700.1	6.6	5.2	7.5	hypothetical protein, conserved
	LdBPK_261610.1	16.0	20.1	30.0	40S ribosomal protein S33, putative (S33-1)

### Functional analysis of the A2 proteins in the CL-SL and VL-SL *L. donovani* strains

**Figure 4:**
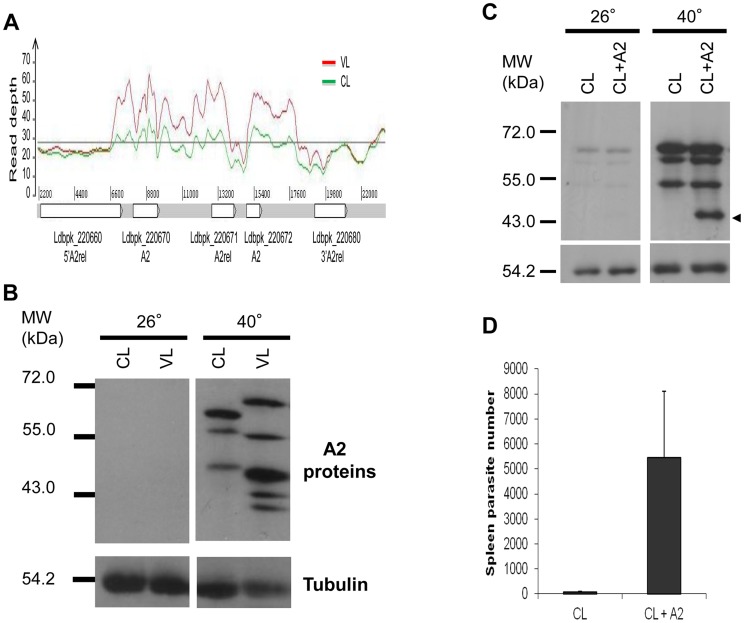
Involvement of A2 in visceral infection. Panel A. Amplification in the region of chromosome 22 containing the A2 gene family. Normalized read coverage for each base position was obtained by dividing its raw read coverage by genome level median coverage (Grey line). Note that there are more A2 gene reads in the VL-SL strain (Red) than from the CL-SL strain (Green). **Panel B.** Comparison of A2 protein expression in the VL-SL and CL-SL strains. A2 expression was induced by 8 h heat shock at 40°C, and A2 proteins (Top panels) and tubulin (Bottom panels) were detected by Western blot. **Panel C.** Ectopically increased expression of A2 in CL-SL. The CL-SL strain was transfected with the pKSneo vector encoding an extra copy of the A2 gene (CL+A2) or the control empty pKSneo vector (CL). A2 proteins (upper panels) and tubulin loading control (lower panels) were detected by Western blot after 4 h at 40°C to induce A2 expression (transfected A2 indicated with an arrow). **Panel D.** Virulence of CL-SL with transfected A2 (CL+A2) was assessed four weeks following intravenous injection (1×10^8^ stationary phase promastigotes) in the tail vein of BALB/c mice (5 mice per group) and spleen parasite burden was determined by limiting dilution of spleen homogenates. Values plus standard error are displayed.

One of the genes with an increased copy number (Table 1) and higher transcript levels (Table 4) in the VL-SL isolate was the A2 gene family. A2 proteins are stress-inducible factors necessary for visceral infection [9], [22]–[24]. We therefore compared sequence reads of the region of chromosome 22 containing several A2 gene family members (see Text S1), and this revealed an amplification of the A2 coding regions in the VL-SL isolate compared to the CL-SL isolate (Fig. 4A). We also performed Western blot analysis to determine whether the increased number of A2 gene sequences resulted in an increase in A2 protein levels. As shown in Figure 4B, there were higher levels of A2 and more A2 protein species in the VL-SL isolate compared to the CL-SL isolate. This observation would be consistent with previous studies showing that A2 genes with greater number of repeat sequences results in larger A2 proteins and are all inducible with heat stress [22]–[24].

Because of the difference in A2 proteins between the VL-SL and CL-SL isolates, we investigated whether this was functionally relevant for the different pathologies displayed by these strains. A2 expression was therefore experimentally increased in the CL-SL strain by transfection with the A2 gene-containing pKSneo plasmid expression vector. This construct was generated from a *L. donovani* genomic library and previously used to characterize the role of the A2 locus in visceralization [13]. Expression of the A2 transgene product (shown with arrow) was effectively up-regulated by temperature stress (40°C) similar to the endogenous chromosomal A2 genes (Fig. 4C). As shown in Fig. 4D, the CL+A2 transfected parasite expressing the additional A2 transgene displayed a higher level of survival in the spleen than the control-transfected CL-SL 4 weeks after infection. It is noteworthy that the increased infection levels in separate experiments ranged from a 5 to 50 fold and were statistically significant revealing that expression of the A2 transgene increased survival in the visceral organs.

We further confirmed the importance of A2 in these clinical isolates by downmodulation of A2 expression in the VL-SL strain and this was associated with impaired survival in the visceral organs (Fig. S3). Overall, these results represent a validation of the importance of A2 in a natural setting and highlight the impact that alterations of A2 levels can have on the parasite's ability to survive in visceral organs. Nevertheless, virulence remained severely attenuated in CL-VL parasites in which A2 expression was partially restored (Fig. 4 C, D) relative to the VL-SL isolate (See also Fig. 1B, C), demonstrating that factors in addition to A2 are required for full virulence in the visceral organs.

## Discussion

A major question concerning leishmaniasis in Sri Lanka is whether one or different *L. donovani* strains are responsible for cutaneous and visceral leishmaniasis. We provide compelling evidence here including fulfilment of Koch's postulates that different strains of *L. donovani* MON-37 are responsible for visceral and cutaneous disease in Sri Lanka. Most strikingly, the CL-SL clinical isolate was severely attenuated for survival in visceral organs in experimentally infected BALB/c mice, yet acquired the ability to cause cutaneous leishmaniasis in humans. Atypical cutaneous leishmaniasis in Sri Lanka caused by *L. donovani* is therefore most likely due to parasite-specific rather than host-specific determinants.

The *L. donovani* VL-SL and CL-SL clinical isolates examined here represent unique strains to investigate genetic determinants affecting disease tropism. There were no homozygous gene deletions and only one pseudogene (containing an additional 3 aa at the C-terminal) specific to the CL-SL strain that were wildtype in the VL-SL strain. SNPs and protein level variations are most likely responsible for disease tropism and pathology differences, and the *Rag C* and *A2* genes identified within are examples of genes which may contribute to the different pathologies caused by the CL-SL and VL-SL strains. It will be of considerable interest to characterize additional CL-SL isolates and determine how widespread changes in these and other genes are on a larger population of parasites from cutaneous leishmaniasis patients in Sri Lanka.

Genome sequencing (Table 1, Fig. 4A) and transcription analysis (Table 4) provided evidence that A2 expression levels were higher in the VL-SL isolate relative to the CL-SL isolate and this was confirmed by Western blot (Fig. 4B). A2 is a multigene family present in *L. donovani* and *L. infantum* but found as pseudogenes in Old World cutaneous leishmaniasis species such as *L. major* and *L. tropica* [22]. Although A2 is present in New World cutaneous *L. mexicana* [3], [25], its size and sequence differ from Old World A2, and therefore may serve a different function. In *L. donovani*, A2 is required for survival in visceral organs and protects *L. donovani* against high temperature (fever) and oxidative stress [23], [24]. As demonstrated within, the expression of a single additional copy of the A2 gene in the CL isolate resulted in increased survival in the spleen. These observations argue that the *L. donovani* CL-SL strain has lost the threshold level of A2 expression necessary for survival in visceral organs and that this represents a major determinant of its attenuation.

It was of interest to identify a non-synonymous SNP in the CL-SL isolate in the *Rag C* gene which in higher eukaryotic cells is involved in the regulation of the mTOR pathway. There are 3 *TOR* genes in *Leishmania* which are all essential for *Leishmania* survival in macrophages or infected mice [20]. Although we did not identify SNPs in the *TOR* genes, this pathway could be compromised by polymorphism in the *Rag C* gene. Expression of the VL-SL *Rag C* gene in the CL-SL isolate significantly increased the ability of the CL-SL isolate to survive in visceral organs (Fig. 2) suggesting that this pathway plays an important role for visceral disease. It has also been demonstrated that targeting the TOR pathway with inhibitors represents a novel opportunity to treat trypanosomatid infections [26]. The observations made here further support this pathway as a drug target for visceral leishmaniasis.

Not only is the *L. donovani* CL-SL strain attenuated for survival in visceral organs, but it has also gained the ability to cause thousands of cutaneous leishmaniasis cases in Sri Lanka. It will be important to identify any gain of function enabling the CL-SL strain to cause cutaneous disease when other naturally-occurring strains of *L. donovani* are unable to do. This may require a better biological readout for the CL-SL *L. donovani* isolate and a better understanding of proteins currently classified as hypothetical.

One of the major observations from recent studies sequencing several different species of *Leishmania* is that although there are relatively few species-specific genes, there is considerably variation in individual gene and chromosome copy numbers [11], [12], [21]. We observed strain-specific differences in the somy of chromosomes 13 and 20 which were trisomic in the VL-SL strain and disomic in the CL-SL strain (Fig. 3A). This lead to an increased level of transcripts from chromosome 20 in the VL-SL isolate relative to the CL-SL isolate though the transcript levels from chromosome 13 are similar in the CL-SL and VL-SL strains (Fig. 3B). This suggests that certain genes encoded on chromosome 20 may play a role in VL-SL pathogenesis. Increased copy number for chromosome 20 would allow for simultaneous amplification and overexpression of these genes. Chromosome 20 encodes 125 hypothetical proteins. Non-hypothetical proteins of interest include glycosomal phosphoglycerate kinase C, one of the five genes with greater than four-fold differences in transcript levels compared to the CL-SL isolate, cytosolic phosphoglycerate kinase B, glycerol-3-phosphate dehydrogenase-like protein, eight calpain-like cysteine peptidases, two DNAj-like protein chaperones, a member of the glutaredoxin antioxidant family, five kinases and three phosphatase subunits. Calpains may play a role in resistance to miltefosine and calpain inhibitors arrest *Leishmania* growth [27].

Overall, this work provides valuable insight into the pathogenesis of visceral leishmaniasis. In particular, gene deletions or pseudogene formation do not appear to be required for *L, donovani* to lose the ability to survive in visceral organs and cause cutaneous disease. The ability of *Leishmania* parasites to cause visceral or cutaneous leishmaniasis may be determined by sequence polymorphisms or amplification of a few genes. This contributes to understanding *L. donovani* virulence and may help to identify intervention targets required for visceral organ infection by this important parasite.

## Materials and Methods

### Ethics statement

Isolation of *L. donovani* from the patients for this study was approved by the ethics review committee of the faculty of Medical Sciences, University of Sri Jayewardenepura, Sri Lanka (approval no 482/09). Written informed consent was obtained prior to parasite isolation from the two adult patients.

All BALB/c mouse infections (permit # 7395) were approved by the McGill University Animal Care Committee following guidelines from the Canadian Council on Animal Care (CCAC).

### Sri Lanka *L. donovani* isolates and culture conditions

*L. donovani* parasites were isolated from two Sri Lankan patients from the Vavuniya district as previously shown geographically [8]. The VL-SL clinical isolate was from a bone marrow aspirate from a 53-year-old male with chronic fever, hepatosplenomegaly, low haemoglobin and seropositivity for rK39 anti-*Leishmania* antibodies as previously described [8]. The CL-SL clinical isolate was derived from a skin lesion on the nose from a 28 year old male. Biopsy samples were directly inoculated into *Leishmania* promastigote culture medium [10], and genomic DNA for genome sequencing was prepared within one month from these promastigote cultures. For axenic amastigote culture, *Leishmania* promastigotes were shifted to 37°C, pH 5.5 culture media to mimic the macrophage phagolysosome environment. Transfections were performed as previously described with the pKSneo-control plasmid, the pKSneo-A2 plasmid or the pKSneo-A2 (R) antisense plasmid [9]. Transfected parasites were maintained in media supplemented with 200 µg/mL G418 (Wisent).

### Infection of BALB/c mice

Female BALB/c mice weighing 17–20 g were purchased from Charles River Breeding Laboratories and maintained in the animal care facility under pathogen-free conditions. BALB/c mice were infected by tail vein injection of stationary-phase promastigotes. Four weeks post infection, amastigotes were isolated from the liver and spleen, and the *Leishmania* parasite number determined by limiting dilution. The number of stationary promastigotes injected is indicated for each experiment in the Figure legends. Liver parasite burdens were also measured by counting the number of amastigotes in Giemsa-stained liver imprints, expressed as Leishman-Donovan Units (LDU): number of amastigotes per 1000 cell nuclei×liver weight [9]. For cutaneous infections, mice were infected subcutaneously with stationary-phase promastigotes in the hind footpads. Disease progression was monitored by weekly caliper measurement of footpad swelling.

### Genomic DNA library preparation, sequencing and analysis

Genomic DNA was sheared into fragments of 100–1,200 bp (Nebulizer, Illumina) and was made into a paired-end DNA sequencing (DNA-seq) library using the Genomic DNA Sample Preparation Kit (Illumina, San Diego, USA). Libraries were sequenced using the Genome Analyzer IIx (Illumina) at the High Throughput Genomics Unit at the University of Washington. Reads were aligned against the reference genome (*L. donovani* BPK282/Ocl4, cloned line from Nepal). Somy and copy number information for each chromosome were calculated independently using custom written perl script entitled “find_copy_number.pl” (see supplementary methods, Text S1). Single nucleotide polymorphism and small indels were called by inputting alignment files (bam) from all the four libraries into GATK software [28]. A thorough manual inspection revealed around 30% of variant calls were false positives or incorrectly genotyped. Calls were then validated manually and reassigned genotypes if necessary. All the validated variants that were consistent within each group (VL-SL and CL-SL) but different between them were then analyzed in detail to study the effects on protein coding genes using SNP EFF(v3.2) tool [29]. Further details concerning genomic library preparation and sequencing are presented in Supplementary Methods (Text S1).

### Total RNA preparation

Promastigote total RNA was extracted from late log phase promastigotes culture. Axenic amastigote total RNA was extracted from axenic amastigote cultures 22 hours after shifting the cells to amastigote culture conditions. B10R macrophages were infected with CL-SL or VL-SL amastigotes at an amastigote/macrophage ratio of 10/1 in suspension in DMEM medium for 12 hours. The percentage of infected macrophages was 72% for CL-SL and 95% for VL-SL; the average amastigotes per infected B10R cell was 3.6 for CL-SL and 3.3 for VL-SL. Infected macrophages were washed and centrifuged to remove extracellular amastigotes and directly suspended in Trizol reagent for total RNA extraction.

### Construction and analysis of Spliced Leader libraries

SL RNA-seq libraries were prepared as previously described [30]. The SL RNA libraries were sequenced using the Genome Analyzer IIx (Illumina) at the High Throughput Genomics Unit at the University of Washington to generate 36-nt long single-end reads. Details of the mapping of high-throughput sequencing reads can be found in the supplementary methods (Text S1). The SL RNA-seq data have been submitted to the GEO database under accession no. GSE48475.

### PCR, expression vector construction, transfection, RT-PCR, SDS-PAGE and western blotting

PCR cloning of VL-SL genes, expression vector constructions and transfection were performed as previously described [10]. To confirm that the corresponding VL-SL genes were expressed in transfected *L. donovani* CL-SL cells, the reverse transcription PCR (RT-PCR) reactions were performed with QIAGEN OneStep RT-PCR Kit. One primer was specific for the transfected VL-SL gene and other primer was specific to the expression vector sequence contained in the 3′ untranslated region (See supplementary Table S4 for primer sequences). 1 µg of total RNA extracted from these transfected CL-SL cells and pretreated with DNase I was used in each RT-PCR reaction. SDS-PAGE and Western blotting to detect A2 was performed as previously described [23]. A2 was detected with a 1∶8,000 dilution of mouse monoclonal anti-A2 antibody and tubulin with a 1∶2,000 dilution of mouse monoclonal anti-tubulin antibody (Oncogene).

### Data access

All information was deposited in GenBank under bioproject ID PRJNA210295. DNA sequencing data can be accessed from the SRA database using accession no. SRS484822 and SRS484824. RNA-seq data has been deposited in the GEO database using accession no SRP026479. Open source software tools we used are referenced from methods section. The locally developed scripts are publicly available to download from github project ‘bifxscripts’ under GPLicence (https://github.com/bifxcore/bifxscripts/tree/master/Ldonovani_tropism)

## Supporting Information

Figure S1 Partial amino acid sequence alignments of the 6-phosphogluconate dehydrogenase (6PGDH) isoenzyme from different *Leishmania* species. *L. donovani* 6PGDH partial amino acid sequences from amino acids 278 to 337 were used for alignments. The main *L. donovani* India (Ld-India) zymodeme (MON-2) sequence is given on top. The previously reported (5) *L. donovani* Sri Lanka (Ld-SrLan) zymodeme (MON-37) is shown below. CL-SL (CL) and VL-SL (VL) are the Sri Lanka *L. donovani* isolates. L. tro, *L. tropica*; L. maj, *L. major*; L. mex, *L. mexicana*; and L. bra, *L. braziliensis* are shown for comparison to other *Leishmania* species. Note: the single amino acid difference at amino acid 326 in red distinguishes the Indian strain (N, asparagine) from the Sri Lanka strain (D, aspartic acid) and the CL and VL isolates are identical with D at amino acid 326.
(DOCX)

Figure S2 Footpad parasite burden is comparable for CL-SL (CL) and VL-SL (VL). Mice were infected subcutaneously in the hind footpad with 5×10^6^ stationary phase promastigotes and sacrificed at five weeks post-infection (at the peak of footpad swelling). Footpad parasite burden was determined by limiting dilution of footpad homogenates. Average values plus standard error for two independent experiments with at least 4 mice/group are shown.
(DOCX)

Figure S3 Down-regulation of A2 in the VL-SL (VL) isolate results in loss of virulence. **Panel A.** Western blot showing down-regulation of A2 in VL by antisense RNA. The VL isolate was transfected with the pKSneo vector encoding antisense A2 RNA that down-regulates A2 protein expression (VL - A2) or the control empty pKSneo vector (VL). A2 proteins (upper panels) and tubulin loading control (lower panels) were detected by Western blot after 4 h at 40°C to induce A2 expression. **Panel B.** Virulence of VL-SL with reduced A2 expression (VL - A2) was assessed four weeks following intravenous injection in the tail vein of BALB/c mice (5×10^7^ stationary phase promastigotes) and spleen parasite burden was determined by limiting dilution of spleen homogenates. Values plus standard error are displayed.
(DOCX)

Table S1 Non-synonymous changes identified in the CL-SL isolate.
(DOCX)

Table S2 Genes with transcript levels more than 2 fold Up-regulated in VL-SL.
(DOCX)

Table S3 Genes with transcript levels more than 2 fold Up-regulated in CL-SL.
(DOCX)

Table S4 List of primers used for RT-PCR.
(DOCX)

Test S1 Supporting methods.
(DOCX)
