# Supplementary material for: Genetic Analysis of Leishmania donovani Tropism Using a Naturally Attenuated Cutaneous Strain
Source: PLoS Pathog. 2014 Jul 3;10(7):e1004244. doi: 10.1371/journal.ppat.1004244 (PMC4081786; doi:10.1371/journal.ppat.1004244)
Supplement: Figure S1 — Partial amino acid sequence alignments of the 6-phosphogluconate dehydrogenase (6PGDH) isoenzyme from different Leishmania species. L. donovani 6PGDH partial amino acid sequences from amino acids 278 to 337 were used for alignments. The main L. donovani India (Ld-India) zymodeme (MON-2) sequence is given on top. The previously reported (5) L. donovani Sri Lanka (Ld-SrLan) zymodeme (MON-37) is shown below. CL-SL (CL) and VL-SL (VL) are the Sri Lanka L. donovani isolates. L. tro, L. tropica; L. maj, L. major; L. mex, L. mexicana; and L. bra, L. braziliensis are shown for comparison to other Leishmania species. Note: the single amino acid difference at amino acid 326 in red distinguishes the Indian strain (N, asparagine) from the Sri Lanka strain (D, aspartic acid) and the CL and VL isolates are identical with D at amino acid 326. (DOCX) [file ppat.1004244.s001.docx]

**Figure S1**

Ld(India) 278 VPAPSLSMAVISRQMTMCKEERIANCKAFPNFPRGPSAEARDKSPNSPNAKQLYHAVSLC 337

Ld(SrLan) ------------------------------------------------D-----------

CL ------------------------------------------------D-----------

VL ------------------------------------------------D-----------

L.tro ------N-------I----------------------E--T-------E-----------

L.maj ------N----------Y-G-----------------E--T-------E--K--------

L.mex ------N----------Y-A--V--S----H--C--CEK-T-------E----F-----S

L.bra ------N------------D--AE-F----T--CSVREQVK-----A-EV---------S

**Figure S1.** Partial amino acid sequence alignments of the 6-phosphogluconate dehydrogenase (6PGDH) isoenzyme from different *Leishmania* species. *L. donovani* 6PGDH partial amino acid sequences from amino acids 278 to 337 were used for alignments. The main *L. donovani* India (Ld-India) zymodeme (MON-2) sequence is given on top. The previously reported (5) *L. donovani* Sri Lanka (Ld-SrLan) zymodeme (MON-37) is shown below. CL-SL (CL) and VL-SL (VL) are the Sri Lanka *L. donovani* isolates. L. tro, *L. tropica*; L. maj, *L. major*; L. mex, *L. mexicana*; and L. bra, *L. braziliensis* are shown for comparison to other *Leishmania* species. Note: the single amino acid difference at amino acid 326 in red distinguishes the Indian strain (N, asparagine) from the Sri Lanka strain (D, aspartic acid) and the CL and VL isolates are identical with D at amino acid 326.
