# Supplementary material for: Genetic Analysis of Leishmania donovani Tropism Using a Naturally Attenuated Cutaneous Strain
Source: PLoS Pathog. 2014 Jul 3;10(7):e1004244. doi: 10.1371/journal.ppat.1004244 (PMC4081786; doi:10.1371/journal.ppat.1004244)
Supplement: Figure S2 — Footpad parasite burden is comparable for CL-SL (CL) and VL-SL (VL). Mice were infected subcutaneously in the hind footpad with 5×10^6^ stationary phase promastigotes and sacrificed at five weeks post-infection (at the peak of footpad swelling). Footpad parasite burden was determined by limiting dilution of footpad homogenates. Average values plus standard error for two independent experiments with at least 4 mice/group are shown. (DOCX) [file ppat.1004244.s002.docx]

**Figure S2**

**Figure S2.** Footpad parasite burden is comparable for CL-SL (CL) and VL-SL (VL). Mice were infected subcutaneously in the hind footpad with 5x10^6^ stationary phase promastigotes and sacrificed at five weeks post-infection (at the peak of footpad swelling). Footpad parasite burden was determined by limiting dilution of footpad homogenates. Average values plus standard error for two independent experiments with at least 4 mice/group are shown.
