# Supplementary material for: Genetic Analysis of Leishmania donovani Tropism Using a Naturally Attenuated Cutaneous Strain
Source: PLoS Pathog. 2014 Jul 3;10(7):e1004244. doi: 10.1371/journal.ppat.1004244 (PMC4081786; doi:10.1371/journal.ppat.1004244)
Supplement: Figure S3 — Down-regulation of A2 in the VL-SL (VL) isolate results in loss of virulence. Panel A. Western blot showing down-regulation of A2 in VL by antisense RNA. The VL isolate was transfected with the pKSneo vector encoding antisense A2 RNA that down-regulates A2 protein expression (VL - A2) or the control empty pKSneo vector (VL). A2 proteins (upper panels) and tubulin loading control (lower panels) were detected by Western blot after 4 h at 40°C to induce A2 expression. Panel B. Virulence of VL-SL with reduced A2 expression (VL - A2) was assessed four weeks following intravenous injection in the tail vein of BALB/c mice (5×10^7^ stationary phase promastigotes) and spleen parasite burden was determined by limiting dilution of spleen homogenates. Values plus standard error are displayed. (DOCX) [file ppat.1004244.s003.docx]

**Figure S3**

**A**

**B**

**Figure S3** Down-regulation of A2 in the VL-SL (VL) isolate results in loss of virulence. **Panel A.** Western blot showing down-regulation of A2 in VL by antisense RNA. The VL isolate was transfected with the pKSneo vector encoding antisense A2 RNA that down-regulates A2 protein expression (VL - A2) or the control empty pKSneo vector (VL). A2 proteins (upper panels) and tubulin loading control (lower panels) were detected by Western blot after 4h at 40°C to induce A2 expression. **Panel B.** Virulence of VL-SL with reduced A2 expression (VL - A2) was assessed four weeks following intravenous injection in the tail vein of BALB/c mice (5x10^7^ stationary phase promastigotes) and spleen parasite burden was determined by limiting dilution of spleen homogenates. Values plus standard error are displayed.
