# Supplementary material for: Genetic Analysis of Leishmania donovani Tropism Using a Naturally Attenuated Cutaneous Strain
Source: PLoS Pathog. 2014 Jul 3;10(7):e1004244. doi: 10.1371/journal.ppat.1004244 (PMC4081786; doi:10.1371/journal.ppat.1004244)
Supplement: Table S1 — Non-synonymous changes identified in the CL-SL isolate. (DOCX) [file ppat.1004244.s004.docx]

**Table S1 Non-synonymous changes identified in the CL-SL isolate**

| **Chrom** | **Position** | **Change** | **Gene ID** | **Size** | **Site** | **Gene product** |
| --- | --- | --- | --- | --- | --- | --- |
| 2 | 143983 | C/A | LdBPK_020280.1 | 690 | G287V | hypothetical protein |
| 3 | 63662 | C/A | LdBPK_030250.1 | 302 | Q44K | hypothetical protein |
| 4 | 233956 | G/A | LdBPK_040560.1 | 1568 | S1355L | hypothetical protein |
| 5 | 187200 | C/T | LdBPK_050580.1 | 779 | P247S | hypothetical protein |
| 6 | 226422 | A/G | LdBPK_060650.1 | 1910 | D179G | hypothetical protein**^n^** |
| 7 | 468732 | T/G | LdBPK_071060.1 | 635 | V101G | hypothetical protein**^n^** |
| 7 | 588612 | C/T | LdBPK_071330.1 | 2542 | R80C | hypothetical protein |
| 8 | 283815 | TCA/- | LdBPK_080670.1 | 1359 | I290- | Protein Kinase |
| 8 | 287023 | G/T | LdBPK_080670.1 | 1359 | R1359L | Protein Kinase |
| 9 | 256960 | C/A | LdBPK_090660.1 | 970 | P315Q | hypothetical protein |
| 12 | 128747 | G/T | LdBPK_120270.1 | 3232 | E1157D | hypothetical protein |
| 13 | 391107 | C/A | LdBPK_131090.1 | 710 | A103S | Adenylosuccinate synthetase |
| 13 | 446304 | C/T | LdBPK_131230.1 | 490 | A376T | hypothetical protein |
| 17 | 184308 | C/G | LdBPK_170470.1 | 543 | G301A | hypothetical protein |
| 17 | 524512 | C/A | LdBPK_171200.1 | 540 | P147Q | 3-oxo-5-alpha-steroid 4-dehydrogenase |
| 20 | 44618 | T/C | LdBPK_200140.1 | 444 | K414R | hypothetical protein |
| 20 | 64115 | C/A | LdBPK_200200.1 | 862 | V536L | hypothetical protein |
| 20 | 378077 | G/A | LdBPK_200960.1 | 699 | G114S | hypothetical protein |
| 21 | 347857 | G/A | LdBPK_211040.1 | 916 | A734T | hypothetical protein**^n^** |
| 21 | 694523 | C/T | LdBPK_211930.1 | 664 | V285M | MFS family protein |
| 22 | 91259 | C/T | LdBPK_220120.1 | 258 | G226D | phosphoinositide phosphatase |
| 22 | 484386 | C/T | LdBPK_221070.1 | 452 | A354T | WD40 repeat protein |
| 22 | 639245 | G/T | LdBPK_221470.1 | 2796 | S1861I | hypothetical protein |
| 23 | 130765 | C/A | LdBPK_230400.1 | 366 | R128S | hypothetical protein |
| 23 | 151670 | G/A | LdBPK_230440.1 | 1983 | A1275T | hypothetical protein |
| 23 | 177737 | G/A | LdBPK_230500.1 | 325 | R20H | trypanothione synthetase, putative |
| 23 | 213708 | T/C | LdBPK_230610.1 | 882 | S531P | hypothetical protein |
| 23 | 764932 | G/T | LdBPK_231940.1 | 965 | Q308H | hypothetical protein |
| 25 | 11450 | C/T | LdBPK_250040.1 | 1267 | E435K | hypothetical protein |
| 25 | 28359 | G/T | LdBPK_250110.1 | 474 | S224R | hypothetical protein |
| 25 | 332157 | G/T | LdBPK_250910.1 | 1332 | D1057Y | hypothetical protein |
| 25 | 354793 | C/T | LdBPK_251000.1 | 1225 | P607S | hypothetical protein |
| 25 | 426490 | C/A | LdBPK_251150.1 | 6525 | A4067S | hypothetical protein |
| 25 | 686731 | G/A | LdBPK_251840.1 | 501 | R270W | hypothetical protein |
| 25 | 813118 | A/G | LdBPK_252290.1 | 377 | S160G | DnaJ family protein |
| 27 | 356907 | C/T | LdBPK_270840.1 | 1879 | A1535T | ABC protein subfamily A, member 9 |
| 29 | 754859 | A/G | LdBPK_291720.1 | 1012 | S936P | hypothetical protein**^n^** |
| 29 | 762074 | T/C | LdBPK_291730.1 | 681 | I529V | hypothetical protein |
| 29 | 887210 | C/T | LdBPK_292100.1 | 1943 | G1406E | hypothetical protein |
| 29 | 888117 | C/T | LdBPK_292100.1 | 1943 | A1104T | hypothetical protein |
| 29 | 890025 | C/T | LdBPK_292100.1 | 1943 | D468N | hypothetical protein |
| 29 | 940243 | C/T | LdBPK_292210.1 | 1602 | S456N | hypothetical protein |
| 29 | 986440 | G/T | LdBPK_292290.1 | 463 | D321E | hypothetical protein |
| 31 | 538009 | TGG/- | LdBPK_311320.1 | 1027 | T352- | hypothetical protein |
| 31 | 690890 | C/T | LdBPK_311500.1 | 1039 | V651I | hypothetical protein**^n^** |
| 31 | 696217 | GAG/- | LdBPK_311510.1 | 1629 | P1496- | hypothetical protein**^n^** |
| 31 | 771837 | C/T | LdBPK_311630.1 | 811 | E676K | hypothetical protein**^n^** |
| 31 | 795383 | G/A | LdBPK_311710.1 | 1438 | S41F | hypothetical protein |
| 31 | 965002 | G/T | LdBPK_311990.1 | 572 | D555E | hypothetical protein |
| 31 | 1005893 | G/A | LdBPK_312080.1 | 500 | P56S | hypothetical protein**^n^** |
| 32 | 130897 | T/G | LdBPK_320370.1 | 366 | K24T | hypothetical protein |
| 32 | 275410 | C/T | LdBPK_320820.1 | 1138 | R854C | protein kinase, putative |
| 32 | 800684 | C/G | LdBPK_322160.1 | 211 | Q163H | ras-related protein rab-2a |
| 32 | 954400 | G/A | LdBPK_322560.1 | 2563 | P1216L | hypothetical protein |
| 32 | 963415 | G/A | LdBPK_322570.1 | 1016 | P80L | hypothetical protein |
| 33 | 203221 | C/T | LdBPK_330640.1 | 190 | R119H | hypothetical protein |
| 34 | 137453 | G/A | LdBPK_340390.1 | 1139 | A955T | hypothetical protein |
| 34 | 678336 | A/G | LdBPK_341580.1 | 1001 | L232S | hypothetical protein |
| 34 | 823181 | C/T | LdBPK_341900.1 | 243 | A50T | NAD dependent deacetylase |
| 34 | 823304 | T/G | LdBPK_341900.1 | 243 | I9L | NAD dependent deacetylase |
| 34 | 1469060 | G/A | LdBPK_343550.1 | 517 | G102D | hypothetical protein |
| 34 | 1511816 | A/C | LdBPK_343690.1 | 4645 | T3702P | dynein heavy chain, putative |
| 35 | 358064 | C/T | LdBPK_350830.1 | 1656 | L688F | hypothetical protein**^n^** |
| 36 | 35955 | G/A | LdBPK_360120.1 | 904 | T286I | hypothetical protein |
| 36 | 403430 | G/A | LdBPK_361070.1 | 1644 | R354K | hypothetical protein |
| 36 | 423688 | C/A | LdBPK_361120.1 | 1007 | P247T | hypothetical protein |
| 36 | 1704662 | C/A | LdBPK_364550.1 | 551 | Q130K | hypothetical protein**^n^** |
| 36 | 2048201 | G/A | LdBPK_365480.1 | 3020 | R2559C | hypothetical protein |
| 36 | 2081812 | T/C | LdBPK_365540.1 | 371 | Q17R | hypothetical protein |
| 36 | 2280763 | G/A | LdBPK_366140.1 | 364 | R231C | ras-like small GTPases, putative |

n Non conserved hypothetical protein.
