# Supplementary material for: Genetic Analysis of Leishmania donovani Tropism Using a Naturally Attenuated Cutaneous Strain
Source: PLoS Pathog. 2014 Jul 3;10(7):e1004244. doi: 10.1371/journal.ppat.1004244 (PMC4081786; doi:10.1371/journal.ppat.1004244)
Supplement: Table S2 — Genes with transcript levels more than 2 fold Up-regulated in VL-SL. (DOCX) [file ppat.1004244.s005.docx]

**Table S2. Genes with transcript levels more than 2 fold Up-regulated in VL-SL**

|  | **Folds change** | | |  |
| --- | --- | --- | --- | --- |
| **Gene ID** | **Am** | **Ax** | **Pro** | **Product Name** |
| LdBPK_221230.1 | 21.0 | 19.1 | 17.2 | hypothetical protein, conserved |
| LdBPK_361170.1 | 5.0 | 3.7 | 3.4 | hypothetical protein, conserved |
| LdBPK_200110.1 | 4.8 | 3.7 | 5.5 | phosphoglycerate kinase C, glycosomal (PGKC) |
| LdBPK_111230.1 | 4.5 | 5.5 | 2.7 | ATP-binding cassette protein subfamily A, member 3, putative |
| LdBPK_220670.1 | 4.3 | 2.5 | 2.4 | A2 protein |
| LdBPK_070420.1 | 3.6 | 2.0 | 2.8 | homoserine dehydrogenase-like protein |
| LdBPK_220100.1 | 3.1 | 2.3 | 1.8 | amino acid permease, putative (AAT2) |
| LdBPK_280540.1 | 3.0 | 0.5 | 0.3 | hypothetical protein, conserved |
| LdBPK_191070.1 | 2.9 | 1.6 | 0.5 | hypothetical protein, unknown function |
| LdBPK_090500.1 | 2.8 | 2.2 | 2.2 | hypothetical protein, conserved |
| LdBPK_150490.1 | 2.7 | 1.7 | 1.2 | hypothetical protein |
| LdBPK_161090.1 | 2.7 | 1.9 | 2.6 | hypothetical protein, unknown function |
| LdBPK_201430.1 | 2.6 | 1.6 | 2.2 | hypothetical protein, conserved |
| LdBPK_111130.1 | 2.5 | 1.2 | 0.6 | 60S ribosomal protein L28, putative |
| LdBPK_161040.1 | 2.5 | 3.0 | 1.9 | hypothetical protein, conserved |
| LdBPK_060310.1 | 2.4 | 4.1 | 5.1 | Folate biopterin transporter, putative |
| LdBPK_161080.1 | 2.3 | 2.4 | 1.4 | hypothetical protein, conserved |
| LdBPK_302960.1 | 2.3 | 1.3 | 1.5 | hypothetical protein, conserved |
| LdBPK_290560.1 | 2.2 | 1.0 | 1.4 | hypothetical protein, conserved |
| LdBPK_090150.1 | 2.2 | 1.3 | 1.5 | kinesin, putative |
| LdBPK_161060.1 | 2.2 | 2.3 | 1.9 | hypothetical protein, conserved |
| LdBPK_161050.1 | 2.2 | 2.2 | 1.6 | hypothetical protein, conserved |
| LdBPK_010690.1 | 2.2 | 2.0 | 1.2 | hypothetical protein, conserved |
| LdBPK_341220.1 | 2.2 | 1.7 | 0.6 | hypothetical protein, conserved |
| LdBPK_050510.1 | 2.2 | 0.6 | 0.7 | ATPase alpha subunit |
| LdBPK_344080.1 | 2.1 | 1.8 | 1.5 | hypothetical protein, conserved |
| LdBPK_120830.1 | 2.1 | 1.1 | 1.2 | puromycin-sensitive aminopeptidase-like protein |
| LdBPK_090290.1 | 2.1 | 1.7 | 1.5 | hypothetical protein, conserved |
| LdBPK_353250.1 | 2.1 | 1.3 | 1.0 | hypothetical protein, conserved |
| LdBPK_200830.1 | 2.1 | 2.0 | 2.3 | hypothetical protein, conserved |
| LdBPK_344090.1 | 2.1 | 0.5 | 0.5 | Unc104-like kinesin, putative |
| LdBPK_212010.1 | 2.1 | 2.5 | 1.9 | protein kinase, putative |
| LdBPK_221080.1 | 2.1 | 1.6 | 1.4 | hypothetical protein, conserved |
| LdBPK_366430.1 | 2.1 | 2.5 | 2.0 | hypothetical protein, conserved |
| LdBPK_010450.1 | 2.1 | 1.2 | 1.5 | hypothetical protein, conserved |
| LdBPK_090640.1 | 2.1 | 1.6 | 1.1 | ATP-dependent DNA helicase, putative |
| LdBPK_090310.1 | 2.1 | 1.7 | 1.5 | hypothetical protein, conserved |
| LdBPK_020700.1 | 2.1 | 2.7 | 0.7 | Dehydrogenase/oxidoreductase-like protein |
| LdBPK_090330.1 | 2.0 | 1.8 | 2.2 | DNA photolyase, putative |
| LdBPK_321980.1 | 2.0 | 5.8 | 3.4 | hypothetical protein, unknown function |
| LdBPK_100180.1 | 2.0 | 1.9 | 10.6 | hypothetical protein, unknown function |
| LdBPK_221360.1 | 2.0 | 1.1 | 1.6 | hypothetical protein, unknown function |
| LdBPK_340190.1 | 2.0 | 1.0 | 1.7 | hypothetical protein |
| LdBPK_090360.1 | 2.0 | 1.9 | 1.6 | hypothetical protein, conserved |
| LdBPK_130680.1 | 2.0 | 1.7 | 1.1 | hypothetical protein, conserved |
| LdBPK_341730.1 | 2.0 | 2.0 | 1.5 | amastin-like surface protein, putative |
| LdBPK_161280.1 | 2.0 | 1.0 | 0.8 | hypothetical protein, conserved |
| LdBPK_361790.1 | 2.0 | 0.8 | 1.1 | phosphatidylinositolN-acetylglucosaminyltransferase subunit c |
| LdBPK_090540.1 | 2.0 | 1.3 | 1.3 | hypothetical protein, conserved |
| LdBPK_090650.1 | 2.0 | 2.2 | 2.4 | cyclin 1, putative,serine peptidase family S51, peptidase E, putative |
| LdBPK_130650.1 | 2.0 | 1.9 | 1.8 | hypothetical protein, conserved |
