# Supplementary material for: Genetic Analysis of Leishmania donovani Tropism Using a Naturally Attenuated Cutaneous Strain
Source: PLoS Pathog. 2014 Jul 3;10(7):e1004244. doi: 10.1371/journal.ppat.1004244 (PMC4081786; doi:10.1371/journal.ppat.1004244)
Supplement: Table S3 — Genes with transcript levels more than 2 fold Up-regulated in CL-SL. (DOCX) [file ppat.1004244.s006.docx]

**Table S3. Genes with transcript levels more than 2 fold Up-regulated in CL-SL**

|  | **Folds change** | | |  |
| --- | --- | --- | --- | --- |
| **Gene ID** | **Am** | **Ax** | **Pro** | **Gene Product** |
| LdBPK_261610.1 | 16.0 | 20.1 | 30.0 | 40S ribosomal protein S33, putative |
| LdBPK_303700.1 | 6.6 | 5.2 | 7.5 | hypothetical protein, conserved |
| LdBPK_365490.1 | 4.7 | 4.4 | 4.5 | adaptin, putative |
| LdBPK_221440.1 | 3.7 | 1.4 | 1.7 | hypothetical protein, conserved |
| LdBPK_250830.1 | 3.3 | 4.5 | 0.5 | hypothetical protein, conserved |
| LdBPK_230290.1 | 3.1 | 2.4 | 4.3 | ABC-thiol transporter (MRPA) |
| LdBPK_302700.1 | 2.8 | 0.4 | 0.9 | hypothetical protein, conserved |
| LdBPK_311860.1 | 2.7 | 2.5 | 2.7 | amino acid permease (AAT8.4) |
| LdBPK_311620.1 | 2.7 | 1.9 | 1.8 | hypothetical protein, conserved |
| LdBPK_242180.1 | 2.6 | 1.4 | 0.6 | hypothetical protein, conserved |
| LdBPK_140530.1 | 2.4 | 3.0 | 1.2 | hypothetical protein, conserved |
| LdBPK_230270.1 | 2.4 | 2.2 | 3.0 | hypothetical protein, conserved |
| LdBPK_323820.1 | 2.3 | 1.1 | 0.9 | 3-hydroxyisobutyryl-coenzyme a hydrolase-like protein |
| LdBPK_230250.1 | 2.3 | 2.1 | 3.3 | hypothetical protein, conserved |
| LdBPK_262270.1 | 2.2 | 3.2 | 0.8 | syntaxin binding protein 1, putative |
| LdBPK_230700.1 | 2.2 | 2.8 | 4.4 | hypothetical protein |
| LdBPK_340920.1 | 2.2 | 2.4 | 1.0 | hypothetical protein, conserved |
| LdBPK_230280.1 | 2.2 | 2.6 | 3.3 | terbinafine resistance locus protein (yip1) |
| LdBPK_333390.1 | 2.2 | 1.6 | 1.1 | h1 histone-like protein |
| LdBPK_131260.1 | 2.2 | 1.2 | 1.8 | hypothetical protein, conserved |
| LdBPK_303420.1 | 2.1 | 1.9 | 1.1 | hypothetical protein, conserved |
| LdBPK_181240.1 | 2.1 | 1.2 | 0.4 | hypothetical protein, conserved |
| LdBPK_292200.1 | 2.1 | 1.0 | 0.9 | hypothetical protein, conserved |
| LdBPK_366560.1 | 2.1 | 1.2 | 1.4 | glucose transporter, lmgt1 |
| LdBPK_261320.1 | 2.1 | 2.5 | 0.9 | DNA ligase k alpha, putative |
| LdBPK_312380.1 | 2.1 | 2.5 | 1.8 | 3’-nucleotidase/nuclease precursor, putative |
| LdBPK_261080.1 | 2.0 | 2.3 | 3.5 | hypothetical protein, conserved |
| LdBPK_303230.1 | 2.0 | 2.5 | 5.0 | 3-hydroxy-3-methylglutaryl-CoA reductase, putative |
| LdBPK_240790.1 | 2.0 | 1.7 | 2.8 | malic enzyme, putative |
| LdBPK_290710.1 | 2.0 | 1.5 | 1.2 | RNA-binding protein, putative |
| LdBPK_262320.1 | 2.0 | 1.3 | 2.2 | hypothetical protein, conserved |
| LdBPK_353950.1 | 2.0 | 1.3 | 0.7 | hypothetical protein |
| LdBPK_260830.1 | 2.0 | 1.4 | 1.4 | hypothetical protein, conserved |
| LdBPK_260230.1 | 2.0 | 1.4 | 2.3 | hypothetical protein, conserved |
| LdBPK_332070.1 | 2.0 | 4.0 | 0.4 | 60S ribosomal protein L37 |
| LdBPK_262100.1 | 2.0 | 1.3 | 1.4 | Serine/threonine protein phosphatase-like protein |
