# Supplementary material for: Genetic Analysis of Leishmania donovani Tropism Using a Naturally Attenuated Cutaneous Strain
Source: PLoS Pathog. 2014 Jul 3;10(7):e1004244. doi: 10.1371/journal.ppat.1004244 (PMC4081786; doi:10.1371/journal.ppat.1004244)
Supplement: Table S4 — List of primers used for RT-PCR. (DOCX) [file ppat.1004244.s007.docx]

**Table S4. List of primers used for RT-PCR**

| Gene ID | DNA Primers | Application | Size (bp) |
| --- | --- | --- | --- |
| LdBPK_171200 | 5’cccaagcttCCAATGGAGATTTGCTTCGT  5’ccgagatctCGAGCGTTTGCCTAAGAGAT | Gene cloning | 1716 |
|  | 5’ATTCCTACTGGGGCTGTGTG  5’CGCAAAGCAATGATCCTCTT* | RT-PCR | 1315 |
| LdBPK_220120 | 5’cccaagctTGTCGAGCTCTGGTATGCTG  5’ccgagatctAGAGTACCCGGTGCAGAGTG | Gene cloning | 871 |
|  | 5’TTCGGCGAATCTCAACAACC  5’CGCAAAGCAATGATCCTCTT* | RT-PCR | 728 |
| LdBPK_252290 | 5’cccaaGCTTGCAGAGTGGAACTCCT  5’ccgagatctCACGCAAACACACCTCACTC | Gene cloning | 1184 |
|  | 5’CAGTGAAAGCGGGTTCTACG  5’CGCAAAGCAATGATCCTCTT* | RT-PCR | 1047 |
| LdBPK_320370 | 5’cccaagcttACACACAAAACGCAAACCTG  5’ccgagatctGGGAACGGGAGGAGAAATAA | Gene cloning | 1213 |
|  | 5’CTTCTCACCGCCTACAGGAC  5’CGCAAAGCAATGATCCTCTT* | RT-PCR | 724 |
| LdBPK_322160 | 5’cccaagctTCTTCTTTTTCCCCACATCG  5’ccgagatctCGTAGGGAGTAAGGCACACC | Gene cloning | 792 |
|  | 5’ CGGCTCATTTCCATCCAAGG  5’CGCAAAGCAATGATCCTCTT* | RT-PCR | 828 |
| LdBPK_341900 | 5’ cccaagcTTTCTCTCACCGCCCTCTTA  5’ccgagatctCAGTTCGGTCCCATCACTTT | Gene cloning | 816 |
|  | 5’CTGGTGCAACCATAATGTCG  5’CGCAAAGCAATGATCCTCTT* | RT-PCR | 872 |
| LdBPK_366140 | 5’cccaagctTCACTACTTGCTCGCCCTTT  5’ccgagatcTTGCGTGCCTCTCTCTCTCT | Gene cloning | 1210 |
|  | 5’CCGTCTTCCAAGCATTCTCG  5’CGCAAAGCAATGATCCTCTT* | RT-PCR | 819 |

*This primer is complementary to the 3’ untranslated region specific to the *Leishmania* expression

vector pLpneo to ensure amplification of the plasmid derived transcripts.

Lower case letters represent restriction enzyme sites used for cloning._._
