# Supplementary material for: Genetic Analysis of Leishmania donovani Tropism Using a Naturally Attenuated Cutaneous Strain
Source: PLoS Pathog. 2014 Jul 3;10(7):e1004244. doi: 10.1371/journal.ppat.1004244 (PMC4081786; doi:10.1371/journal.ppat.1004244)
Supplement: Test S1 — Supporting methods. (DOCX) [file ppat.1004244.s008.docx]

**Supplementary Methods**

6PGDH DNA sequencing

The 6-phosphogluconate dehydrogenase (6PGDH) gene was PCR amplified with primers (6PGDH-F: AATCGAGCAGCTCAAGGAAG and 6PGDH-R:GAGCTTGGCGAGAATCTGAC) as previously described [[1](#_ENREF_1)]. The resulting 997 bp 6PGDH PCR product was cloned into the TA cloning vector (Invitrogen) and subjected to Sanger DNA sequencing at the McGill University Genome Quebec Innovation Centre.

**Genomic DNA library preparation and Sequencing**

Two types of genomic DNA libraries, paired and single-end, were constructed from the DNA of each isolate. Genomic DNA was sheared into fragments of 100–1,200 bp (Nebulizer, Illumina) and paired-end libraries constructed using the Genomic DNA Sample Preparation Kit (Illumina, San Diego, USA). Paired-end libraries were sequenced using the Genome Analyzer IIx (Illumina) at the Covance Inc., Seattle, to generate 100-nt long paired-end reads. Single-end libraries were sequenced using the Genome Analyzer IIx (Illumina) at the High Throughput Genomics Unit at the University of Washington to generate 36-nt long single-end reads. The reads were checked for their overall quality and GC content using FastQC tool (http://www.bioinformatics.babraham.ac.uk/projects/fastqc/); reads with average quality value less than 30 were removed. The overall GC content of the reads was checked to be similar to that of *Leishmania donovani* (~59.5%). Reads with Illumina adapter sequences were trimmed off using cutadapt (v1.2) software (<http://journal.embnet.org/index.php/embnetjournal/article/view/200>). *L. donovani* (BPK282/Ocl4, cloned line from Nepal) reference genome sequences were obtained from Welcome Trust Sanger Institute's ftp site (<ftp://ftp.sanger.ac.uk/pub/pathogens/Leishmania/donovani/>). Paired-end reads were aligned against the reference genome using BWA (version 0.7.3a-r367) [[2](#_ENREF_2)], configured to allow a maximum of two mismatches (-k 2) in a 23 bases long seed sequence (-l 23) and its *sampe* module, with expected maximum insert size of 1000 bases (-a 1000), was used to generate the alignments in sam format. The default values were accepted for all other BWA parameters. Single-end reads were aligned using *samse* module with similar alignment stringency. SAMtools (v0.1.18) was used to convert sam files into binary format (samtools view), sort (samtools sort) and index (samtools index) bam files.

**Chromosome Somy**

Somy for chromosomes in each library was calculated independently. An in-house built perl script entitled “find_copy_number.pl”, was used to calculate somy. The median coverage for each of the chromosomes was first calculated, then a median of all chromosomal medians was determined and divided by two which represents the median coverage for a haploid allele of a chromosome. Then median coverage of each chromosome was then divided by the haploid chromosome coverage to obtain somy of individual chromosomes. Median values of each chromosome in each of four libraries were subjected to T-test to identify chromosomes with significant change in somy.

**Copy number variations**

Copy number variation within each chromosome was calculated using two complementary approaches. In the first approach we calculated copy number of each gene from each library. using read coverage from coding regions only. A normalized copy number for each “gene” was obtained by dividing the median coverage of the coding region with median coverage of the entire chromosome. The obtained values were then subjected to a T-test to identify gene segments with different copy number between VL-SL and CL-SL replicates. This approach however also identified a number of false positives. Therefore, a second approach was used and involved a manual inspection of somy corrected read coverage across the chromosome. In this approach, each chromosome is divided into 10,000 base non-overlapping tiles. The copy numbers of the tiles were calculated by dividing the median read coverage by the median read coverage of the entire chromosome. The copy numbers across the chromosomes were manually examined to identify the regions where both VL-SL libraries collinearly differ from both CL-SL libraries. Though, this approach identified copy number variation comprehensively, it alone lacks statistical support. Therefore, we intersected the results from gene centric approach with visual coverage analysis to identify the most probable genes and regions of chromosome that have copy number variations.

**Variant Prediction**

The RealingerTargetCreater script from the GATK suite [[4](#_ENREF_4)] was used to identify all intervals from bam files that contained indels. Then IndelRealigner script extracted reads from these regions and performed a local alignment (smith-waterman based) creating a new bam file with realigned indel regions. Variants were called by inputting alignment files (bam) from all the libraries together into the UnifideGenotyper script from the GATK suite. Both single nucleotide polymorphisms and small indels were called simultaneously using the –genotype_likelihood_models=BOTH option. Identification of high quality variants was enforced by (a) restricting our identification to regions with a combined coverage of 250 (all 4 libraries; -dcov=250), (b) bases with a phred-scaled quality score of 21 or more (-min_base_quality_score=21), (c) variants with a phred-scaled confidence of at least 30, (-stand_emit_conf=30) and (d) --sample_ploidy=8. The variants with phred-scaled confidence between 30 and 60 marked as LowQual (-stand_call_conf=60) were carefully validated by manual inspection. A thorough manual inspection revealed around 30% of variant calls were false positives or incorrectly genotyped. Most of these false predictions were located in the regions of low coverage. We subsequently went through the coverage, nucleotide frequencies, genotype likelihood values etc., for each variant in each sample to validate the calls and reassign genotypes if necessary. For example, if a SNP was genotyped as homozygous reference in VL-SL and heterozygous in CL-SL, the coverage across all the libraries was examined to ensure coverage was uniform across the region and second ensure minimum coverage of 25 reads from each of the libraries The PL values of the CL-SL samples were checked for the probabilities of that being any genotype other than the called one. If the p-value for that being homozygous reference was less than 0.05, then the genotype to be homozygous reference was assigned. Variants from each position of the genome were then checked to see if they are consistent within both samples of VL-SL and consistent within both samples of CL-SL but are different between VL-SL and CL-SL group. Only those satisfying this criterion were selected for testing their effect on the proteome and manually reviewed in the IGV browser. We used the LdBPK reference genome annotations from GeneDB and snpEFF(v3.2)[[5](#_ENREF_5)] tool to identify variants location and the effect it might confer on the protein coding abilities of the gene.

**Mapping of high-throughput SL-RNA sequencing reads**

Reads containing a TTG tag at the 5' end, indicating the presence of authentic SL sequences, were separated from non–TTG-containing reads. The first three bases (TTG) of the reads were trimmed off, and the remaining sequence was aligned against the reference genome *L. donovani* BPK282 using bowtie [[6](#_ENREF_6)] which is configured to allow a maximum of one mismatch in 15 bases long seed region (-n 1; -l 15) and sum of the phred-quality values at all mismatched positions was kept under 70 (-e 70). The post alignment analysis revealed only <3% of the aligned reads have mismatches. While >80% of them had only one mismatch, none of the reads had more than 2 mismatches. Alignments in sam files were converted to sorted and indexed binary bam files using SAMtools [[3](#_ENREF_3)] utility. “A set of internally developed Perl scripts (”bam2count_slmap3.pl” and “count_slresults_gene_slsite.pl”) were used to calculate the number of reads aligned against positive strand of each gene region (which includes the CDS and the 5' upstream intergenic region). EdgeR software [[7](#_ENREF_7)] was used for differential expression analysis of count data from all six cDNA libraries.

**A2 Gene Analysis**

The A2 locus on chr22 is misassembled (and poorly annotated) in the LdBPK reference sequence, due to the complicated repetitive nature of this locus.  Indeed, this is the case for all current *Leishmania* genomes except LmjF, where the locus was sequenced from individual cosmid clones. Thus, we corrected the LdBPK (and LinJ) reference sequences by manual reconstruction of the A2 locus using the LmjF sequences as a guide.  We first identified the region of the LdBPK reference sequence that corresponded to the A2 locus that is present as an inverted repeat on either side of a divergent strand switch region in LmjF and added an additional copy of this sequence to replace the sequences gaps in the existing LdBPK reference sequence, as well as realigning some of the sequences contigs within the region between inverted repeats.  Structural and functional annotation of this region was also corrected manually.  This process resulted in the reconstructed A2 locus that contained two copies of the A2 gene in each inverted repeat unit, as well as a single copy of 3’A2rel, A2rel and 5’A2rel in each repeat unit.  While this is almost certainly not the correct sequence of the locus in either the VL or CL genome (as evidence by variation in the read coverage plots in Figure 4A), it did now allow us to distinguish the difference in A2 gene content between the isolates that is clear from the Western blot analysis in Figure 4B.

**Supplementary references**

1. Ranasinghe S, Zhang WW, Wickremasinghe R, Abeygunasekera P, Chandrasekharan V, et al. (2012) Leishmania donovani zymodeme MON-37 isolated from an autochthonous visceral leishmaniasis patient in Sri Lanka. Pathogens and global health 106: 421-424.

2. Li H, Durbin R (2010) Fast and accurate long-read alignment with Burrows-Wheeler transform. Bioinformatics 26: 589-595.

3. Li H, Handsaker B, Wysoker A, Fennell T, Ruan J, et al. (2009) The Sequence Alignment/Map format and SAMtools. Bioinformatics 25: 2078-2079.

4. DePristo MA, Banks E, Poplin R, Garimella KV, Maguire JR, et al. (2011) A framework for variation discovery and genotyping using next-generation DNA sequencing data. Nat Genet 43: 491-496.

5. Cingolani P, Platts A, Wang LL, Coon M, Nguyen T, et al. (2012) A program for annotating and predicting the effects of single nucleotide polymorphisms, SnpEff: SNPs in the genome of Drosophila melanogaster strain w(1118); iso-2; iso-3. Fly 6: 80-92.

6. Langmead B, Trapnell C, Pop M, Salzberg SL (2009) Ultrafast and memory-efficient alignment of short DNA sequences to the human genome. Genome biology 10: R25.

7. Robinson MD, McCarthy DJ, Smyth GK (2010) edgeR: a Bioconductor package for differential expression analysis of digital gene expression data. Bioinformatics 26: 139-140.
